# A New Deep-Learning Method for Human Activity Recognition

**DOI:** 10.3390/s23052816

**Published:** 2023-03-04

**Authors:** Roberta Vrskova, Patrik Kamencay, Robert Hudec, Peter Sykora

**Affiliations:** Department of Multimedia and Information-Communication Technologies, University of Zilina, 010 26 Zilina, Slovakia

**Keywords:** deep learning, 3DCNN, ConvLSTM, human activity recognition

## Abstract

Currently, three-dimensional convolutional neural networks (3DCNNs) are a popular approach in the field of human activity recognition. However, due to the variety of methods used for human activity recognition, we propose a new deep-learning model in this paper. The main objective of our work is to optimize the traditional 3DCNN and propose a new model that combines 3DCNN with Convolutional Long Short-Term Memory (ConvLSTM) layers. Our experimental results, which were obtained using the LoDVP Abnormal Activities dataset, UCF50 dataset, and MOD20 dataset, demonstrate the superiority of the 3DCNN + ConvLSTM combination for recognizing human activities. Furthermore, our proposed model is well-suited for real-time human activity recognition applications and can be further enhanced by incorporating additional sensor data. To provide a comprehensive comparison of our proposed 3DCNN + ConvLSTM architecture, we compared our experimental results on these datasets. We achieved a precision of 89.12% when using the LoDVP Abnormal Activities dataset. Meanwhile, the precision we obtained using the modified UCF50 dataset (UCF50mini) and MOD20 dataset was 83.89% and 87.76%, respectively. Overall, our work demonstrates that the combination of 3DCNN and ConvLSTM layers can improve the accuracy of human activity recognition tasks, and our proposed model shows promise for real-time applications.

## 1. Introduction

Presently, there is an increased emphasis on safety, and video capture and storage devices are constantly evolving to meet this demand. However, these devices need to be equipped with a system capable of accurately classifying various abnormal incidents and reducing human error. By increasing the effectiveness of surveillance systems, we could not only reduce crime and prevent various incidents but also provide first aid as soon as possible. Currently, video classification research in the field of computer vision has become a popular yet challenging topic. In addition to static information, videos also contain time information. Therefore, it is necessary to take into account the previous and subsequent frames to accurately recognize and classify incidents. This makes incident recognition from video more challenging than image recognition. In recent years, 3DCNN (3D Convolutional Network) and ConvLSTM (Convolutional Long Short-Term Memory) networks have emerged as popular techniques for video classification. These models are capable of capturing both spatial and temporal features, enabling them to accurately classify videos with high accuracy. Overall, with the continued development and improvement of video classification techniques, we can create more effective surveillance systems that can enhance safety and security in various settings.

In [1], the authors employ a combination of ConvLSTM and Conv3D layers for the task of human activity recognition. In [2], the authors use 3DCNN networks for Facial Micro-Expression recognition. Gesture recognition is covered in [3], where the classic 3DCNN network and the 3D Resnet network are used. In [4], the authors propose several approaches for the classification of abnormalities in video, including a deep hierarchical architecture that extracts generic video descriptors using 3DCNN and BICLSTM layers. As part of this work, they also use a combination of 3DCNN, ConvLSTM, and Conv2D layers. In [5], the authors develop a non-contact method of assessing respiratory rate in sub-optimal light using video recordings, combining Euler scaling and a 3DCNN network to eliminate the need for Region of Interest (ROI). In [6], the authors develop a framework that takes pose-based skeleton joint sequences as input, followed by an LSTM network to learn the temporal evolution of the poses. The obtained results are compared with two fine-tuned deep neural networks, ConvLSTM and 3DCNN, and the Histogram of the Optical Flow (HOF) descriptor achieves the best results when used with the MLP classifier. This approach is applied by the authors in the diagnosis of Autism Spectrum Disorder (ASD) using video recordings, which is also covered in [7]. In [7], the authors use popular neural networks, 3DCNN and ConvLSTM, to detect diseases from video. In [8], the authors propose a hierarchical LSTM Convolutional Neural Network for the classification of farmers’ behavior in agriculture. Finally, gesture recognition is also dealt with in [9], where the authors use two types of neural networks, 3DCNN and ConvLSTM network [10,11,12,13].

In our previous work, we also focused on the classification and recognition of video incidents. In [14,15], we proposed 3DCNN and ConvLSTM neural network approaches, respectively. For the recognition of abnormal incidents, we created the LoDVP Abnormal Activities database [14]. We trained and tested the proposed ConvLSTM network on this database, achieving an accuracy of 96.19%. The proposed 3DCNN neural network was trained and tested on the UCF YouTube action, UCF50, and UCF101 databases, achieving accuracy values of 87.4%, 80.6%, and 78.5%, respectively. It is worth noting that the proposed ConvLSTM network in [14] was specifically designed for our LoDVP Abnormal Activities database, which may have contributed to its high accuracy. Meanwhile, the proposed 3DCNN network in [15] was tested on several well-known databases, which demonstrated its generalizability to different video recognition tasks.

Based on the results we have obtained thus far, as well as the current state of the field, we have decided to focus on combining ConvLSTM and 3DCNN networks. Specifically, we aim to leverage both ConvLSTM layers and Conv3D layers in our approach. By doing so, we hope to achieve better performance in video recognition tasks, as both types of layers have shown promise in previous work. We believe that this approach has the potential to yield improved accuracy and generalizability in video recognition tasks.

## 2. Materials and Methods

In our research, we are investigating a combination of 3DCNN and ConvLSTM networks for video classification. ConvLSTM networks use their temporal memory to capture spatiotemporal patterns in videos, while 3DCNN networks leverage the third dimension for classification. Both networks are widely used for video and image classification in various fields including industry and medicine.

### 2.1. 3DCNN Architecture

**Figure 1:**
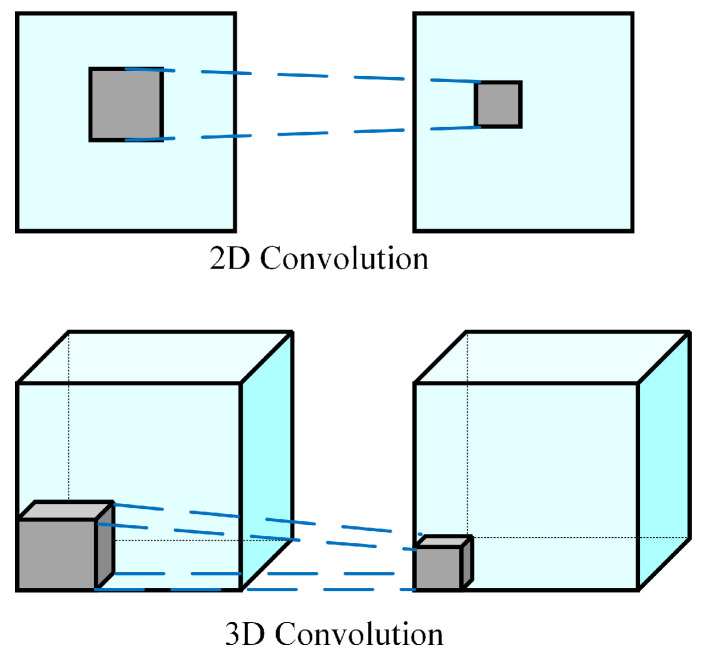
Comparison of the mathematical operation of 2D convolution and 3D convolution [16].

The 3DCNN neural network can analyze and identifying different moving 2D objects in images and 3D images, such as in medical imaging. In 3DCNN, the 3D convolution operation is applied to the dataset in three directions (x, y, z) using a three-dimensional filter as is shown in Figure 1. The values in the layer within the three-dimensional filter must be set to be non-negative. The equation below defines the value for each position in the 3D convolution map of features in the layer:

(1) $$v_{ij}^{xyz}=\tanh\langle b_{ij}+\sum_{m}\sum_{p=0}^{P_{i}-1}\sum_{q=0}^{Q_{i}-1}\sum_{r=0}^{R_{i}-1}w_{ijm}^{pqr}v_{\langle i-1\rangle m}^{\langle x+p\rangle \langle y+q\rangle \langle z+r\rangle },$$

where $w_{ijm}^{pqr}$ expresses the value of the kernel attached to the convolutional feature map in the previous layer, $R^{i}$ expresses the size of the 3D kernel [15].

The 3D convolution is created by stacking adjacent layers around the center of the cube, and the convolution maps are interconnected, which captures motion information. However, the convolutional kernel can only extract one type of feature. Generally, 3DCNN is similar to Conv2D (2D Convolutional Neural Network). Combining multiple convolutional layers can improve the results of 3DCNN, similar to 2D convolution. When constructing a 3DCNN, it is crucial to set the number of layers, the number of filters in each layer, and the filter size properly. If pooling is used in the neural network design, the pooling size must have three dimensions to accommodate the 3D data. The output shape from the 3DCNN network is a 3D volume space [16,17,18].

### 2.2. ConvLSTM Architecture

**Figure 2:**
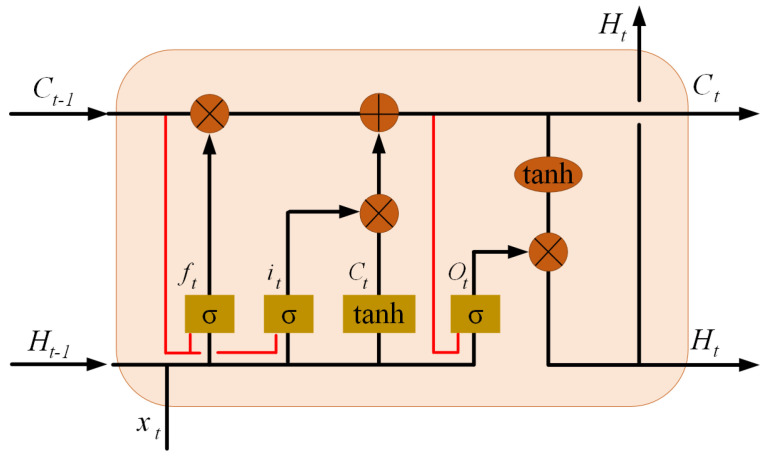
Inner structure of ConvLSTM [19].

The ConvLSTM neural network was developed by combining a Convolutional Neural Network (CNN) and a Long Short-Term Memory (LSTM) network. The ConvLSTM network is similar to an LSTM network in that it is a memory network, but it performs convolution operations on the transitions between layers. The internal design of a ConvLSTM network is illustrated in Figure 2 [19].

The ConvLSTM neural network is commonly used for time-dependent image and video recognition, as it is equipped to capture spatial and temporal correlations. ConvLSTM implements a convolutional operation on the transitions between states and inputs. If we view states as hidden representations of moving objects, a ConvLSTM with a larger transition kernel can capture faster motions, while a network with a smaller kernel can capture slower motions. The key equations of ConvLSTM are derived from LSTM equations by convolutional coupling, as shown below, where “*” denotes the convolution operator and “∘” denotes the Hadamard product:

(2) $$\begin{array}{c} i_{t}=\sigma \left(W_{xi}*X_{t}+W_{hi}*H_{t-1}+W_{ci}\circ C_{t-1}+b_{i}\right), \end{array}$$

(3) $$\begin{array}{c} f_{t}=\sigma \left(W_{xf}*X_{t}+W_{hf}*H_{t-1}+W_{cf}\circ C_{t-1}+b_{f}\right), \end{array}$$

(4) $$\begin{array}{c} C_{t}=f_{t}\circ C_{t-1}+i_{t}\circ tanh\left(W_{xc}*X_{t}+W_{hc}*H_{t-1}+b_{f}\right), \end{array}$$

(5) $$\begin{array}{c} o_{t}=\sigma \left(W_{xo}*X_{t}+W_{ho}*H_{t}+W_{co}\circ C_{t-1}+b_{o}\right), \end{array}$$

(6) $$\begin{array}{c} H_{t}=o_{t}\circ tanh\left(C_{t}\right), \end{array}$$

where cell inputs are labeled $X_{t}$ and cell states are labeled $C_{t}$, hidden states are labeled $H_{t}$; the gates are $i_{t},f_{t},o_{t}$ and σ is a sigmoidal function. We denote the convolution kernels in the equation $W_{-}$ [19].

### 2.3. Proposed 3DCNN + ConvLSTM Architecture

**Figure 3:**
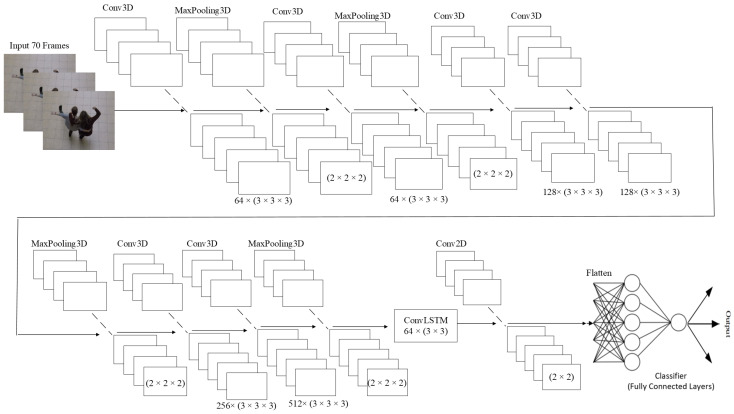
Proposed 3DCNN + ConvLSTM architecture.

Our proposed neural network architecture combines Conv3D layers with a ConvLSTM network layer and a Conv2D layer. The architecture, referred to as 3DCNN + ConvLSTM, contains multiple Conv3D layers followed by a single ConvLSTM layer and a single Conv2D layer. The design of the architecture is depicted in Figure 3. This proposed architecture comprises the following layers:

- Conv3D layers: These layers extract spatiotemporal features from the input video data. The number of Conv3D layers can be adjusted based on the complexity of the task. These layers incorporate a three-dimensional filter, which performs convolution by moving in three directions (x, y, z).
- MaxPooling3D layer is a mathematical operation for 3D data (reduction of 3D data).
- ConvLSTM layer: This layer processes the extracted features from the Conv3D layers and captures the temporal dependencies between the frames.
- Conv2D layer is layer, which applies convolution on 2D data. This layer performs the final classification based on the output of the previous layers.
- A flatten layer converts the output matrix to the vector.

**Figure 4:**
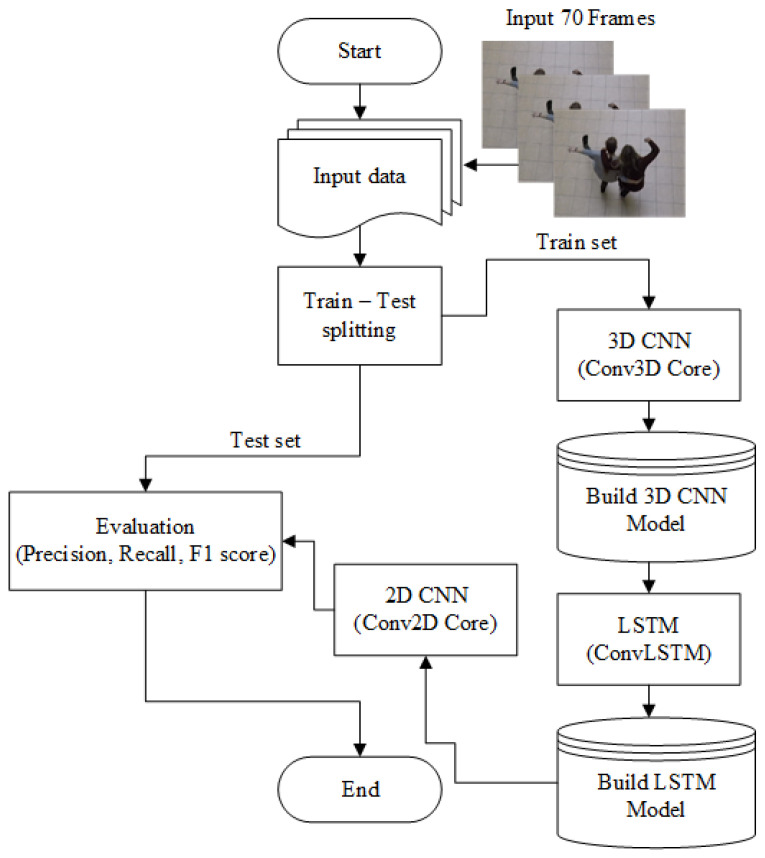
The flowchart of the proposed architecture.

The proposed architecture combines the strengths of both Conv3D and ConvLSTM networks. This architecture consists of multiple 3D convolutional layers, a single ConvLSTM layer, and a single 2D convolutional layer, as well as batch normalization, a flattened layer, and a dense layer. The 3D convolutional part of the architecture was adopted from a previous study [15], while the ConvLSTM part was based on another previous research [14]. The hyperparameters of the 3D convolutional layers and MaxPooling, such as the number of filters and kernel size, are determined by mathematical constraints, with the output of the Conv3D layer being constrained to non-negative integer values. The flowchart of the proposed architecture is illustrated in Figure 4.

**Figure 5:**
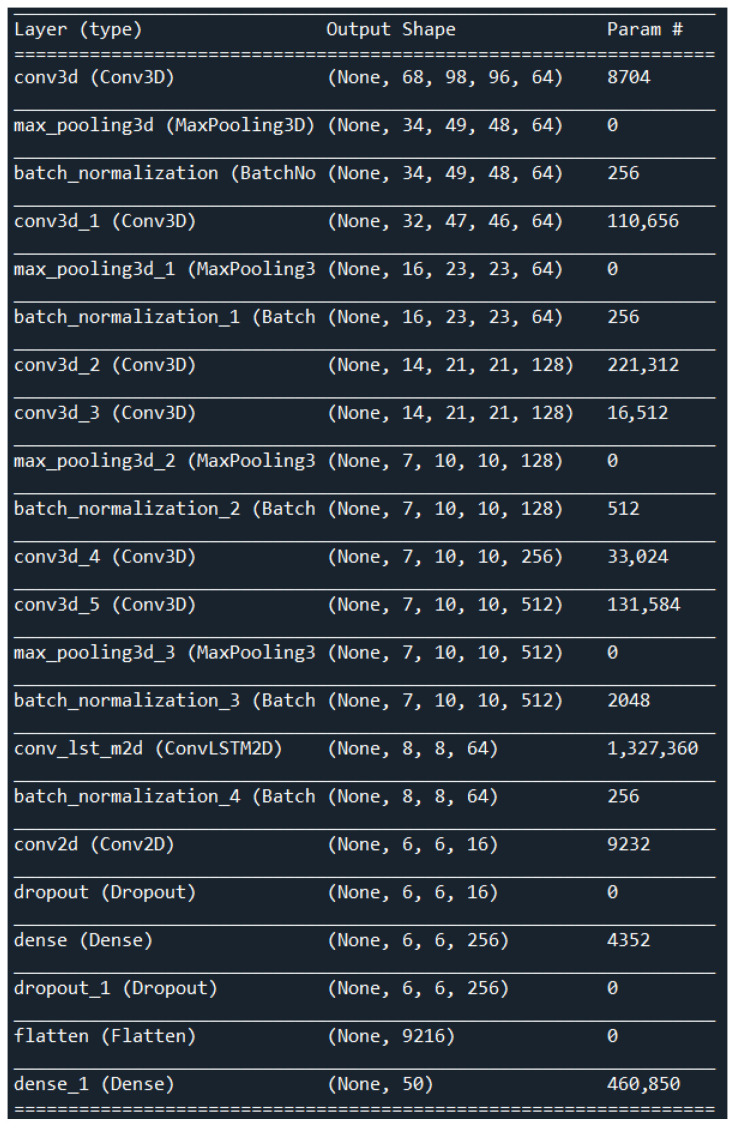
Description of the layers our proposed architecture.

The proposed 3DCNN + ConvLSTM architecture in this work consists of six 3D convolution layers, four MaxPooling3D layers, and one ConvLSTM layer followed by a single Conv2D layer. The input to the network has dimensions of 100 × 100 × 3 (width, height, and number of channels). The first 3D convolution layer uses 64 filters with a kernel size of 3 × 3 × 3. Following each of these 3D convolution layers are MaxPooling3D layers of size 2 × 2 × 2 with a stride of 2. The next two 3D convolution layers have 128 filters of size 3 × 3 × 3, and after these two layers, there is a MaxPooling3D layer of size 2 × 2 × 2. The last two 3D convolution layers have 256 and 512 filters of size 3 × 3 × 3, respectively, and are used in the 3D convolution part of the network. The entire 3DCNN network has a batch normalization layer after every MaxPooling3D layer to improve the training process. The ConvLSTM network includes one ConvLSTM layer with a size of 3 × 3 and 64 filters. After the ConvLSTM layer, there is a batch normalization layer and a Conv2D layer with 16 filters of size 2 × 2. The output of the Conv2D layer is then passed through a flattened layer that turns a matrix into a vector, and the final dense layer has only one neuron that directly predicts the class of the input. The used optimization algorithm was “Adamax” and the learning rate was set to 0.001. A detailed description of the layers of the proposed 3DCNN + ConvLSTM architecture is shown in Figure 5.

The values in Table 1 show the total number of parameters, as well as the number of trainable and non-trainable parameters.

Deep-learning systems are built using Python libraries, such as Keras and TensorFlow, and the experimental results were obtained using the Nvidia CUDA libraries. The input data consisted of images with dimensions of 100 × 100 and 3 channels. For each database, 70% of the data were used for training, 20% for testing, and 10% were reserved for validation (in a 70:20:10 split).

## 3. Description of the Datasets

In this section, the used datasets will be described. The all experimental results on the LoDVP Abnormal Activities dataset, UCF50 dataset, and MOD20 dataset were obtained.

### 3.1. UCF50 Dataset

UCF50 dataset consisted of 50 action categories. The dataset included realistic videos from YouTube. The dataset had large variations in camera motion, cluttered backgrounds, illumination conditions, etc.

**Figure 6:**
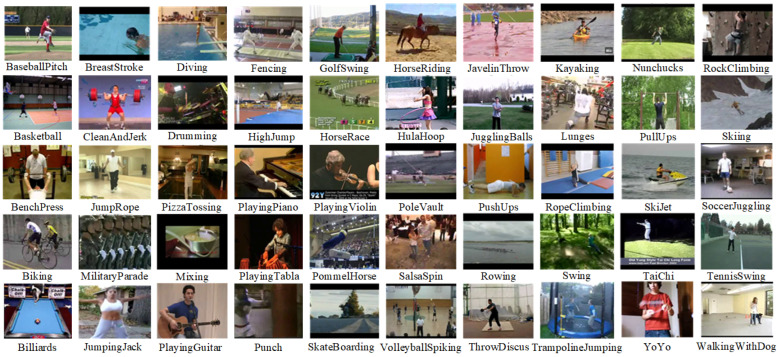
Example of the UCF50 dataset [20].

Videos in the same group may share some common features, such as the same person, similar background, similar viewpoint, etc. The UCF50 dataset can be seen in Figure 6. The UCF50 dataset consisted of 50 categories. The dataset consists of categories such as Baseball Pitch, Basketball Shooting, Bench Press, Biking, Billiards Shot, Breaststroke, Clean and Jerk, Diving, Drumming, Fencing, Golf Swing, Playing Guitar, High Jump, Horse Race, Horse Riding, Hula Hoop, Javelin Throw, Juggling Balls, Jump Rope, Jumping Jack, Kayaking, Lunges, Military Parade, Mixing Batter, Nun chucks, Playing Piano, Pizza Tossing, Pole Vault, Pommel Horse, Pull Ups, Punch, Push Ups, Rock Climbing Indoor, Rope Climbing, Rowing, Salsa Spins, Skate Boarding, Skiing, Skijet, Soccer Juggling, Swing, Playing Tabla, TaiChi, Tennis Swing, Trampoline Jumping, Playing Violin, Volleyball Spiking, Walking with a Dog, and Yo Yo [20].

We cut the dataset used into 10 layers and used both datasets in training the network to compare the results. We called the reduced dataset UCF50mini. Both datasets (reduced and total) were divided into three sets (training, test and validation set).

### 3.2. LoDVP Abnormal Activities Dataset

The LoDVP Abnormal Activity dataset comprises 1069 videos. The incidents in the videos are created by non-professional actors. The videos in the database are created believably. Incidents are reported in the parking lot, in the university campus and in the forest. The scenes were recorded from different angles. The dataset is divided into 11 classes, and each class contains about 100 videos. The length of the video depends on the incident and lasts from 1 s to 30 s. Similar videos belonging to the same class may exhibit common traits, such as a recurring individual, perspective of the camera, and a comparable setting [14].

**Figure 7:**
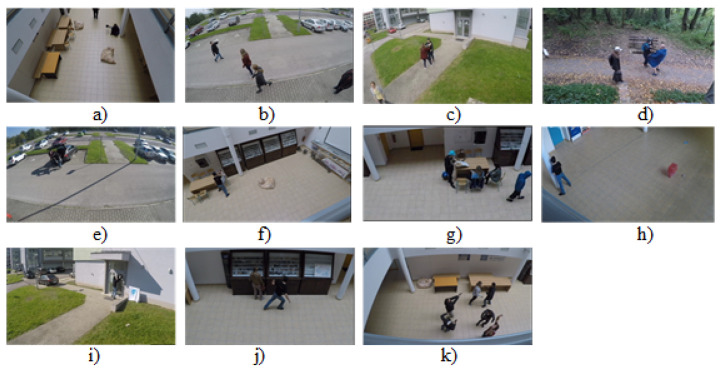
LoDVP Abnormal Activities dataset: (**a**) Begging (**b**) Drunkenness (**c**) Fight (**d**) Harassment (**e**) Hijack (**f**) Knife hazard (**g**) Normal videos (**h**) Pollution (**i**) Property damage (**j**) Robbery (**k**) Terrorism [14].

The LoDVP Abnormal Activity dataset consists of the following classes, which can be seen in Figure 7. For our work, this dataset was also divided into a training test and a validation set in the same ratio as UCF50mini. The division was in the ratio of 70:20:10.

### 3.3. MOD20 Dataset

**Figure 8:**
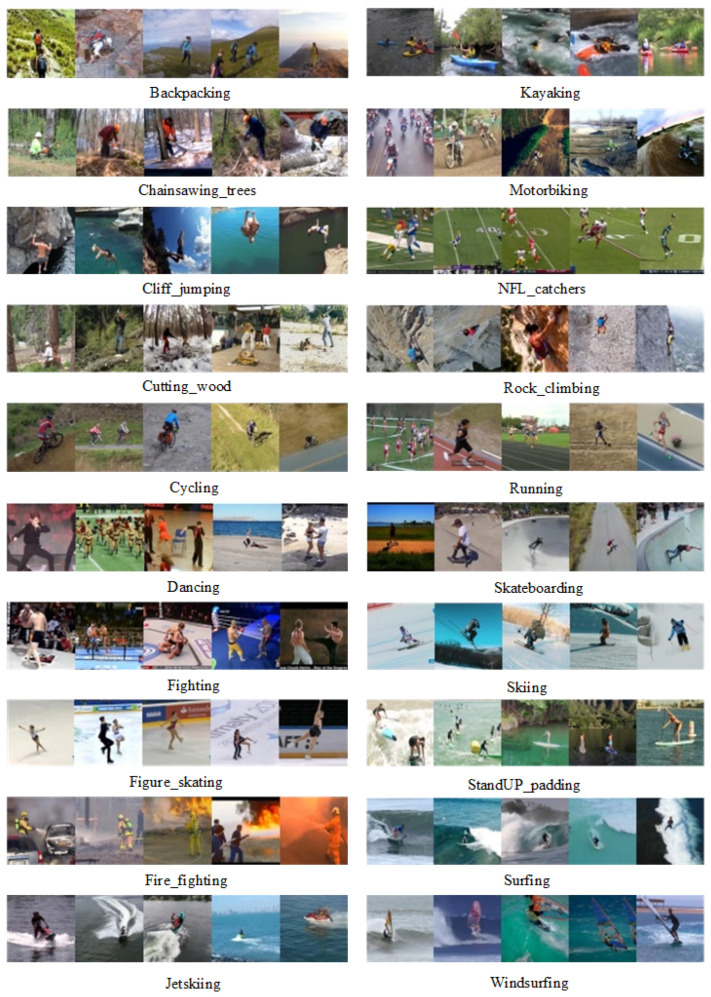
The example of the MOD20 dataset [21].

The MOD20 dataset consists of 2324 videos, of which six videos were created by a quadrotor UAV and 2318 videos were downloaded from YouTube. All clips are 1:1 aspect ratio. Videos were sampled below 29.97 fps. The videos in the dataset are recorded from both fixed and moving cameras. The videos show realistic scenarios in 20 selected classes (see Figure 8). The dataset consists of classes such as backpacking, cliff jumping, cutting wood, cycling, dancing, fighting, figure-skating, fire-fighting, chainsawing trees, jet skiing, kayaking, motorbiking, football-catching, rock-climbing, running, skateboarding, skiing, standup-padding, surfing, and windsurfing [21].

## 4. Experimental Results

This section describes the experimental results achieved on the LoDVP Abnormal Activities dataset, UCF50 dataset, and MOD20 dataset, which demonstrate the superiority of the 3DCNN + ConvLSTM combination in recognizing human activities. To provide a more comprehensive comparison of the proposed architecture, we compared the experimental results achieved on these datasets. In our work, we divided this dataset into a training, testing, and validation set in the same ratio as the UCF50 mini and LoDVP Abnormal Activities datasets, with a data distribution of 70:20:10.

### Results

**Figure 9:**
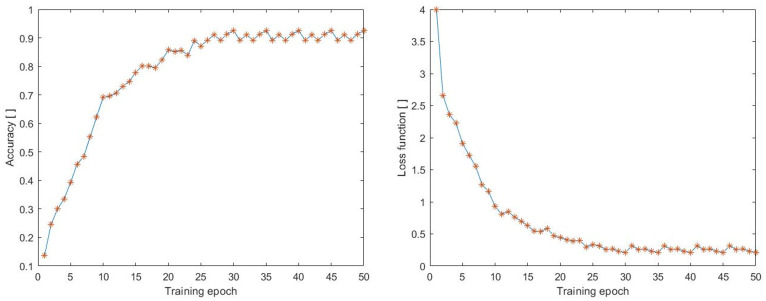
Accuracy during training process on the dataset (LoDVP Abnormal Activities).

All of the tested datasets, including LoDVP Abnormal Activities, UCF50, and UCF50 mini, were divided into three main parts: training set, testing set, and validation set. In this study, the first step was to classify the LoDVP Abnormal Activities dataset into 11 classes, which included Begging, Drunkenness, Fight, Harassment, Hijack, Knife Hazard, Normal Videos, Pollution, Property Damage, Robbery, and Terrorism. The data underwent preprocessing, which involved resizing each video to 100 × 100 size with 70 frames. The accuracy and loss functions during training are displayed in Figure 9.

In the early epochs, we observe a gradual increase in accuracy, and the highest accuracy achieved during training is 92.5%. At the same time, there is a decrease in the loss function, which is directly proportional to the increase in accuracy during the training process. The accuracy and loss function have a directly proportional relationship, and the lowest achieved loss function value during the training process was 0.2106.

**Figure 10:**
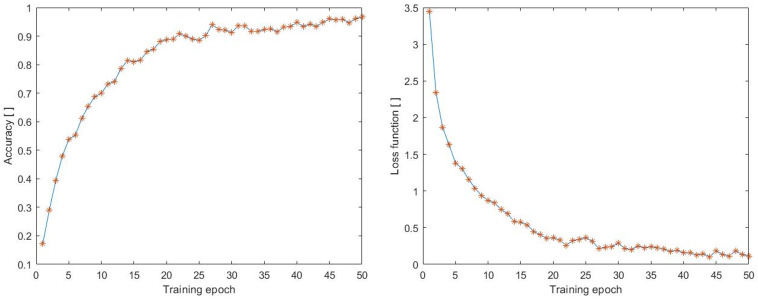
Accuracy during training process on the dataset (UCF50mini).

To provide a comparison, we also monitored the accuracy and loss function achieved on the UCF50mini dataset during the training process. The UCF50mini dataset includes 10 classes: Baseball Pitch, Basketball Shooting, Bench Press, Biking, Billiards Shot, Breaststroke, Clean and Jerk, Diving, Drumming, and Fencing. The data were preprocessed in the same way as in the previous case, with a size of 100 × 100 and 70 frames. The accuracy and loss function can be seen in Figure 10, which displays an increase in accuracy and a decrease in loss function similar to the previous case. The highest accuracy attained was 0.9668, and the lowest loss function value during training was achieved by the neural network architecture with a value of 0.1042.

**Figure 11:**
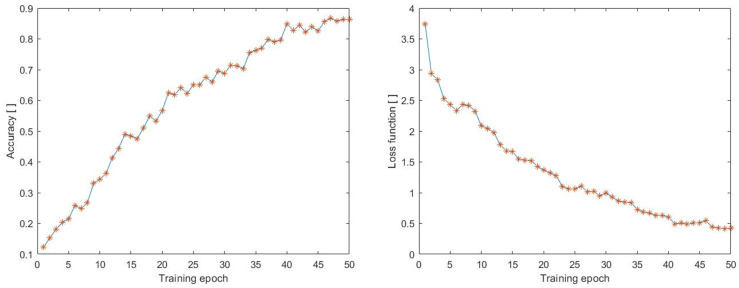
Accuracy during training process on the dataset (MOD20).

We also monitored the training process for comparison with the MOD20 dataset and focused on accuracy and loss function (see Figure 11). The MOD20 dataset consists of 20 classes (with backpacking, cliff jumping, cutting wood, cycling, dancing, fighting, figure-skating, fire-fighting, chainsawing trees, jet skiing, kayaking, motorbiking, football-catching, rock-climbing, running, skateboarding, skiing, standup-padding, surfing, and windsurfing). The data were preprocessed in the same way as in the previous cases, with a size of 100 × 100 and 70 frames. Figure 11 shows a linear increase in accuracy and a decrease in loss function, as observed previously. The highest accuracy value achieved was 0.8630, while the smallest value of the loss function was 0.4223. However, compared to the other datasets, we achieved the worst results during the training process on this dataset.

To make a clearer comparison, we created Table 2, which shows the training and testing results of our proposed model on all three datasets, namely LoDVP Abnormal Activities, UCF50, and UCF50mini. The table presents the values of training loss and training accuracy achieved during the training process, as well as the test loss and test accuracy obtained on a separate set of data. We computed the test results by evaluating the trained model on the test set, which was not used for training or validation. The Table 2 provides a comprehensive comparison of the performance of our proposed model on all three datasets, allowing us to evaluate its generalization ability and robustness to different activity categories.

In both cases, we evaluated the confusion matrix for the LoDVP Abnormal Activities and UCF50mini datasets. The first confusion matrix is shown in Table 3, which expresses the results of the neural network architecture tests on the LoDVP Abnormal Activities dataset. All classes were divided into the following categories: 1. Begging, 2. Drunkenness, 3. Fight, 4. Harassment, 5. Hijack, 6. Knife hazard, 7. Normal videos, 8. Pollution, 9. Property damage, 10. Robbery, 11. Terrorism. The confusion matrix shows how the tested videos were correctly and incorrectly classified into the given categories. For most classes, the proposed architecture did not have a classification problem. However, for the third class (Fight), we observe an increased error rate, where four videos were misclassified into the first class (Begging) and six videos into the second class (Drunkenness). These errors may have occurred due to the similarity of the videos. For example, sometimes a small fight can be confused with begging, and begging can turn into a fight. The similarity between a fight and drunkenness is also quite high, as drunk people can push each other violently, which can lead to a fight.

Furthermore, we created a confusion matrix to display the results of testing the proposed neural network architecture on the UCF50mini dataset (see Table 4). The classes in the confusion matrix are divided into the following categories: 1. Baseball Pitch, 2. Basketball Shooting, 3. Bench Press, 4. Biking, 5. Billiards Shot, 6. Breaststroke, 7. Clean and Jerk, 8. Diving, 9. Drumming, 10. Fencing. The confusion matrix shows the increased accuracy during classification within the testing process. Upon observing the confusion matrix, we can assess that the biggest problem in the classification occurred in category two Basketball Shooting, where four videos were incorrectly classified into category four Biking. The neural network architecture also had a problem classifying class seven Clean and Jerk, where it misclassified one video into class three Bench Press and two videos into the Biking class.

Similarly, we evaluated the confusion matrix for the UCF50mini dataset, which is shown in Table 4. The dataset consists of ten classes, and the confusion matrix shows how the tested videos were correctly and incorrectly classified into these classes. The proposed architecture performed well for most classes, with only a few misclassifications. However, the model had difficulty distinguishing between two classes, namely Billiards Shot and Drumming. Some videos were misclassified as Billiards Shot when they should have been Drumming, and vice versa. This could be due to the similarity in the movements of the two activities, such as hand-eye coordination and rhythmic movements.

We also created a confusion matrix to display the results of testing the neural network architecture on the MOD20 dataset (see Table 5). In the confusion matrix, the classes are assigned numbers as follows: 1. tourism, 2. cliff jumping, 3. chopping wood, 4. cycling, 5. dancing, 6. fighting, 7. figure-skating, 8. Fire-fighting, 9. motorized saw-trees, 10. jet ski, 11. kayak, 12. motorcycle, 13. FOOTBALL-catching, 14. rock-climbing, 15. running, 16. skateboarding, 17. skiing, 18. stand-up paddling, 19. surfing, and 20. windsurfing. The confusion matrix enables us to monitor the accuracy of classification within the testing process. By observing the confusion matrix, we can evaluate that there is not one class with the biggest problem with classification, but the problems within the classification are evenly distributed. The biggest mistakes made in placing a video into a class were two videos. However, we can state that the best results were achieved by categories 13 (FOOTBALL-catching), 18 (stand-up paddling), and 20 (windsurfing), where the neural network architecture had no problem with classification. The unproblematic classification of the mentioned classes may be due to the clarity of the activity occurring in the video.

In general, we have evaluated the results of our tests in several tables. First, we assessed the resulting values of metrics such as F1 score, Precision, and Recall across all three datasets (see Table 6). We can observe that the best results using the proposed architecture were achieved on the UCF50mini dataset. However, the metric results were roughly similar across all three datasets.

Moreover, we compared the performance of our proposed architecture with other available architectures. We applied the architectures to the given datasets and compared their accuracy values. In Table 7, we can observe the resulting accuracy value after testing on the LoDVP Abnormal Activities dataset. We tested ConvLSTM architectures published in the article [21] and 3D Resnet networks such as 3D Resnet50, 3D Resnet101, and 3D Resnet152 [22] on the same dataset. From these results, we can see that our proposed architecture, which combines 3DCNN with ConvLSTM, has the third-best value. Therefore, we can evaluate that our architecture can classify various abnormal incidents such as harassment, fight, etc. very well compared to other architectures. However, compared to classical ConvLSTM, it did not achieve the best results.

In Table 8, we can observe the accuracy results of the same neural network architectures as in the previous case, i.e., ConvLSTM and 3D Resnet50, 101, 152, tested on the UCF50mini dataset. In this case, our proposed architecture achieved significantly better results than the other architectures after the testing process. Our 3DCNN + ConvLSTM architecture achieved an accuracy of 87.7%.

Furthermore, we trained and tested the MOD20 dataset with our proposed architecture and compared the results with the Kernelized Rank-Based Pooling (KRBP) and Feature Subspace-Based Kernelized Rank Pooling (KRP-FS) approaches used by the authors [21]. The best results were achieved using the 3DCNN + ConvLSTM architecture, with an accuracy of 78.21%. The accuracy results of our proposed neural network architecture and the aforementioned approaches are shown in Table 9.

Based on the results obtained on the three datasets (LoDVP Abnormal Activities, UCF50mini, and MOD20), we can conclude that combining 3DCNN and ConvLSTM layers can lead to a neural network architecture whose results are comparable to or better than other available approaches. In terms of experimental results, we compared the performance of our proposed architecture on the LoDVP Abnormal Activities and UCF50mini datasets with architectures such as ConvLSTM and 3D Resnet50,101,152. In addition, we compared the accuracy values obtained on the MOD20 dataset with the BKRP and KRP-FS approaches. Our proposed architecture achieved an accuracy of 89.41% on the LoDVP Abnormal Activity dataset, 87.78% on the UCF50mini dataset, and 78.21% on the MOD20 dataset.

## 5. Conclusions and Future Work

This paper proposes a mixed-architecture neural network for classifying human activities from videos. The architecture combines a 3DCNN network layer and a ConvLSTM layer. We trained and tested our network on three databases: UCF50mini (where we selected the first 10 classes), MOD20, and LoDVP Abnormal Activity. To ensure fair comparison between the datasets, we reduced the UCF50 database to match the number of classes in the other two datasets. For UCF50mini, we performed classification on 10 classes: Baseball pitch, Basketball shooting, Bench press, Cycling, Billiard shooting, Breaststroke, Pure movement, Diving, Drumming, and Fencing. For LoDVP Abnormal Activities, we classified 11 classes: begging, drunkenness, fighting, harassment, kidnapping, knife danger, common videos, pollution, property damage, robbery, and terrorism. MOD20 is the largest dataset with 20 classes: tourism, cliff jumping, logging, cycling, dancing, fighting, figure-skating, fire-fighting, chainsawing trees, water skiing, kayaking, motorcycle, football-catching, climbing, running, skateboarding, skiing, standup-paddling, surfing, and windsurfing. The input videos in all datasets were cropped to 100 × 100 × 3 RGB, and we used 70 frames as input for the neural network architecture.

The results showed that the combined 3DCNN + ConvLSTM neural network was effective in classifying video data containing various human activities. The training on the UCF50mini dataset resulted in a decrease in the loss function to 0.2106 and an increase in accuracy to 92.50%. For the LoDVP Abnormal Activities dataset, the loss function decreased to 0.1042 and accuracy increased to 96.68% during training. On the MOD20 dataset, the loss function during training was 0.4223 and accuracy increased to 86.30%. When comparing the results on the datasets during testing, the combined architecture coped well with the problem of temporal continuity between images. The confusion matrix across all three datasets showed that the classification process was successful, with minimal errors in the average of each class. The overall accuracy of the UCF50 mini dataset test was 87.78%, with precision of 87.76% and recall of 88.63%. The F1 score was 87.84%. For the LoDVP Abnormal Activity dataset, the overall accuracy was 93.41%, with precision of 89.12%, recall of 87.69%, and F1 score of 89.32%. On the MOD20 dataset, the overall accuracy was 78.21%, with precision of 83.89%, recall of 81.09%, and F1 score of 81.57%. The results showed that the 3DCNN + ConvLSTM neural network is capable of classifying video data containing various human activities, with high accuracy and minimal errors in the average of each class. The proposed architecture achieved good results when compared to other existing networks designed for video-based human behavior classification. Overall, the results demonstrate the success in creating a neural network architecture combining 3DCNN and ConvLSTM layers for classifying human behavior in videos.

However, we aim to continue our work and improve the classification results of human activities captured in videos. Accurate classification of human behavior by neural networks can significantly enhance their practical applications. To provide a more comprehensive evaluation of our network’s performance, we compared it to other available neural network architectures, such as 3D ResNet 50,101,152, ConvLSTM, KRBP, and KRP-FS approaches, using three different datasets. Our contribution focuses primarily on recognizing and classifying non-standard human behavior in public spaces, which has a significant impact on the scientific community. The proposed 3DCNN + ConvLSTM architecture has wide-ranging applications in fields such as security and medicine and is comparable to existing networks designed for video-based human behavior classification. However, monitoring and detecting unusual behavior in public places such as city parks and squares is still a challenging task, and our proposed combination of 3DCNN and ConvLSTM has some limitations, including:

- Limited interpretability: 3DCNN with ConvLSTM is a deep-learning architecture, and like most deep-learning models, it is not transparent in how it makes predictions (understanding how the model arrives at a particular decision can be challenging).
- Limited availability of training data: The training of 3DCNN with ConvLSTM requires a large amount of high-quality data to produce good results. This can be a significant limitation in many applications where such data are not readily available.
- Difficulty in tuning hyperparameters: 3DCNN with ConvLSTM involves several hyperparameters that need to be tuned correctly to achieve optimal performance. Tuning these hyperparameters can be time-consuming and requires a significant amount of expertise and experimentation.
- Sensitivity to noise and missing data: The combination of 3DCNN and ConvLSTM relies on the temporal coherence of data for accurate predictions. Therefore, the model can be sensitive to noise and missing data in the input, which can significantly affect the model’s performance.

In summary, the proposed combination of 3DCNN with ConvLSTM is a powerful deep-learning architecture with several limitations, which can impact its scalability, interpretability, data requirements, hyperparameter tuning, and sensitivity to noise and missing data.

In the future work, we plan to explore the incorporation of additional sensor data, such as depth cameras and inertial measurement units, to enhance the performance of the proposed model. We also plan to investigate the use of transfer learning techniques to adapt the model to different domains and environments. Furthermore, we aim to investigate the use of the proposed model for other related tasks such as anomaly detection. Finally, we will also investigate the possibility of deploying the model on edge devices for real-time monitoring. We believe that incorporating additional sensor data could further improve the performance of our model and we look forward to exploring this direction in future work.

## Figures and Tables

**Table 1:** The overall number of parameters of our suggested architecture.

Parameters of the Proposed Architecture	Number of Parameters
Total parameters	2,326,914
Trainable parameters	2,325,250
Non-Trainable parameters	1664

**Table 2:** The accuracy and loss function of the model over 50 epochs, during both training and testing phases.

Evaluation Metrics	MOD20	UCF50mini	LoDVP Abnormal Activities
Train loss	0.4223	0.2106	0.1042
Train accuracy	86.30%	92.50%	96.68%
Test loss	0.5614	0.3568	0.3982
Test accuracy	78.21%	87.78%	83.12%

**Table 3:** The example of the confusion matrix for LoDVP Abnormal Activities.

Targeted/ Predicted	1	2	3	4	5	6	7	8	9	10	11
1	16	0	0	0	0	0	0	0	0	0	0
2	0	14	2	0	0	0	0	0	0	0	0
3	4	6	18	0	0	0	0	0	0	0	0
4	0	0	0	16	0	0	4	0	0	0	0
5	0	0	0	0	20	0	0	0	0	0	0
6	0	2	0	0	0	8	0	0	0	0	0
7	0	0	0	2	0	0	18	2	0	0	0
8	0	0	0	6	0	0	2	12	0	0	0
9	0	0	0	0	0	0	0	0	26	0	0
10	2	0	0	0	0	0	0	0	0	4	0
11	0	0	0	0	0	0	0	0	0	0	14

**Table 4:** The example of the confusion matrix for UCF50mini.

Targeted/ Predicted	1	2	3	4	5	6	7	8	9	10
1	14	0	0	1	0	0	0	0	0	0
2	0	11	0	4	0	0	0	0	0	0
3	0	0	5	0	0	0	0	0	1	0
4	1	1	0	13	0	0	0	1	0	1
5	0	0	0	0	15	0	0	0	0	0
6	0	0	0	0	0	8	0	0	0	0
7	0	0	1	2	0	0	9	0	0	0
8	0	0	0	0	1	0	0	12	0	0
9	0	0	1	0	0	0	0	0	17	0
10	0	0	0	0	0	0	1	0	1	11

**Table 5:** The example of the confusion matrix for MOD20.

Targeted/ Predicted	1	2	3	4	5	6	7	8	9	10	11	12	13	14	15	16	17	18	19	20
1	9	0	0	1	0	0	0	0	0	2	0	0	0	1	2	0	0	0	0	1
2	0	9	0	2	0	0	1	0	0	0	0	0	1	0	0	0	0	0	0	0
3	1	0	10	0	0	0	0	0	0	1	0	0	0	0	1	0	0	1	0	0
4	0	0	0	7	1	0	0	1	0	0	0	1	0	0	0	0	0	0	0	0
5	2	0	0	0	6	0	2	0	0	1	0	0	0	2	0	0	0	0	0	1
6	1	0	0	2	0	6	0	0	0	0	0	1	0	0	0	0	1	0	0	0
7	0	0	0	0	0	0	14	0	0	0	0	0	1	0	0	0	0	0	0	0
8	0	0	0	0	0	0	0	14	0	0	0	0	0	0	0	0	0	2	0	0
9	0	0	0	1	0	2	0	0	7	0	0	0	0	0	1	0	0	0	0	1
10	1	0	1	0	0	0	0	0	1	9	0	0	0	1	0	0	0	0	0	0
11	0	0	0	0	0	0	0	0	0	0	11	0	0	0	0	1	0	1	0	0
12	0	0	0	0	0	0	0	0	0	0	0	12	2	0	0	0	0	0	0	0
13	0	0	0	0	0	0	0	0	0	0	0	0	11	0	0	0	0	0	0	0
14	1	0	0	0	0	0	0	0	0	0	0	0	1	9	0	0	0	0	1	0
15	1	0	0	0	0	0	0	0	0	0	0	0	0	0	10	0	0	0	0	0
16	0	0	0	0	0	0	0	0	0	0	1	0	0	0	0	11	0	0	0	0
17	0	0	2	0	0	0	0	0	0	0	0	0	0	0	0	0	9	0	0	0
18	0	0	0	0	0	0	0	0	0	0	0	0	0	0	0	0	0	14	0	0
19	0	0	0	0	0	0	0	0	0	0	0	0	0	1	0	0	0	1	12	0
20	0	0	0	0	0	0	0	0	0	0	0	0	0	0	0	0	0	0	0	11

**Table 6:** The evaluation criterion of the proposed neural network architectures using different datasets.

Evaluation Metrics	MOD20	UCF50mini	LoDVP Abnormal Activities
Precision (P)	83.89%	87.76%	89.12%
Recall (R)	81.09%	88.63%	87.69%
F1 score (F1)	81.57%	87.84%	89.32%

**Table 7:** Accuracy comparison of the proposed architecture with various neural network architectures using the LoDVP dataset for detecting abnormal activities.

Video Recognition Architectures	Accuracy [%]
Proposed architecture	93.41
ConvLSTM [21]	92.38
3D Resnet50 [22]	36.19
3D Resnet101 [22]	61.90
3D Resnet152 [22]	90.48

**Table 8:** Accuracy comparison of the proposed architecture with various neural network architectures using the UCF50mini dataset.

Video Recognition Architectures	Accuracy [%]
Proposed architecture	87.78
ConvLSTM [21]	80.38
3D Resnet50 [22]	71.53
3D Resnet101 [22]	75.91
3D Resnet152 [22]	83.39

**Table 9:** Accuracy comparison of the proposed architecture with various neural network architectures using the MOD20 dataset.

Video Recognition Architectures	Accuracy [%]
Proposed architecture	78.21
BKRP [21]	66.55
KRP-FS [21]	74.00

## Data Availability

The data presented in this study are available on request from the corresponding author. This is according to the laboratory rules.

## Abbreviations

ConvLSTM	Convolutional Long Short-Term Memory
3DCNN	3D Convolutional Network
KRP-FS	Feature Subspace-Based Kernelized Rank Pooling
BKRP	Kernelized Rank-Based Pooling
BILSTM	Bidirectional Long Short-Term Memory
Conv2D	2D Convolutional Neural Network
Conv3D	3D Convolutional Neural Network
ROI	Region of Interest
MLP	Multilayer perceptron
HOF	Histogram of oriented gradients
ASD	Autism Spectrum Disorder
